# The effects of COVID-19 on cognitive performance in a community-based cohort: a COVID symptom study biobank prospective cohort study

**DOI:** 10.1016/j.eclinm.2023.102086

**Published:** 2023-07-21

**Authors:** Nathan J. Cheetham, Rose Penfold, Valentina Giunchiglia, Vicky Bowyer, Carole H. Sudre, Liane S. Canas, Jie Deng, Benjamin Murray, Eric Kerfoot, Michela Antonelli, Khaled Rjoob, Erika Molteni, Marc F. Österdahl, Nicholas R. Harvey, William R. Trender, Michael H. Malim, Katie J. Doores, Peter J. Hellyer, Marc Modat, Alexander Hammers, Sebastien Ourselin, Emma L. Duncan, Adam Hampshire, Claire J. Steves

**Affiliations:** a Department of Twin Research and Genetic Epidemiology, King's College London, London, United Kingdom; b Edinburgh Delirium Research Group, Ageing and Health, Usher Institute, University of Edinburgh, Edinburgh, United Kingdom; c Department of Brain Sciences, Imperial College London, United Kingdom; d MRC Unit for Lifelong Health and Ageing, Department of Population Health Sciences, University College London, London, United Kingdom; e Centre for Medical Image Computing, Department of Computer Science, University College London, London, United Kingdom; f School of Biomedical Engineering & Imaging Sciences, King's College London, London, United Kingdom; g Department of Infectious Diseases, King's College London, London, United Kingdom; h Centre for Neuroimaging Sciences, King's College London, London, United Kingdom; i King's College London & Guy's and St Thomas' PET Centre, King's College London, London, United Kingdom; j Guy's & St Thomas's NHS Foundation Trust, London, United Kingdom

**Keywords:** Cognition, Cognitive impairment, COVID-19, SARS-CoV-2, Long COVID, COVID-19 recovery

## Abstract

**Background:**

Cognitive impairment has been reported after many types of infection, including SARS-CoV-2. Whether deficits following SARS-CoV-2 improve over time is unclear. Studies to date have focused on hospitalised individuals with up to a year follow-up. The presence, magnitude, persistence and correlations of effects in community-based cases remain relatively unexplored.

**Methods:**

Cognitive performance (working memory, attention, reasoning, motor control) was assessed in a prospective cohort study of participants from the United Kingdom COVID Symptom Study Biobank between July 12, 2021 and August 27, 2021 (Round 1), and between April 28, 2022 and June 21, 2022 (Round 2). Participants, recruited from the COVID Symptom Study smartphone app, comprised individuals with and without SARS-CoV-2 infection and varying symptom duration. Effects of COVID-19 exposures on cognitive accuracy and reaction time scores were estimated using multivariable ordinary least squares linear regression models weighted for inverse probability of participation, adjusting for potential confounders and mediators. The role of ongoing symptoms after COVID-19 infection was examined stratifying for self-perceived recovery. Longitudinal analysis assessed change in cognitive performance between rounds.

**Findings:**

3335 individuals completed Round 1, of whom 1768 also completed Round 2. At Round 1, individuals with previous positive SARS-CoV-2 tests had lower cognitive accuracy (N = 1737, β = −0.14 standard deviations, SDs, 95% confidence intervals, CI: −0.21, −0.07) than negative controls. Deficits were largest for positive individuals with ≥12 weeks of symptoms (N = 495, β = −0.22 SDs, 95% CI: −0.35, −0.09). Effects were comparable to hospital presentation during illness (N = 281, β = −0.31 SDs, 95% CI: −0.44, −0.18), and 10 years age difference (60–70 years vs. 50–60 years, β = −0.21 SDs, 95% CI: −0.30, −0.13) in the whole study population. Stratification by self-reported recovery revealed that deficits were only detectable in SARS-CoV-2 positive individuals who did not feel recovered from COVID-19, whereas individuals who reported full recovery showed no deficits. Longitudinal analysis showed no evidence of cognitive change over time, suggesting that cognitive deficits for affected individuals persisted at almost 2 years since initial infection.

**Interpretation:**

Cognitive deficits following SARS-CoV-2 infection were detectable nearly two years post infection, and largest for individuals with longer symptom durations, ongoing symptoms, and/or more severe infection. However, no such deficits were detected in individuals who reported full recovery from COVID-19. Further work is needed to monitor and develop understanding of recovery mechanisms for those with ongoing symptoms.

**Funding:**

10.13039/100011721Chronic Disease Research Foundation, 10.13039/100010269Wellcome Trust, 10.13039/501100000272National Institute for Health and Care Research, 10.13039/501100000265Medical Research Council, 10.13039/501100000274British Heart Foundation, 10.13039/501100000320Alzheimer's Society, European Union, COVID-19 Driver Relief Fund, French National Research Agency.

**Research in context**

**Evidence before this study**

Abstracts were screened from a PubMed search query (COVID-19) AND (long COVID) AND (cognitive impairment), which returned 409 results between 2020 and January 20, 2023. Multiple systematic reviews and meta-analyses reported consistent observation of cognitive deficits following SARS-CoV-2 infection. Most studies of cognitive impairment have used small samples of less than 200 participants (including any controls), hospitalised cohorts, and measured cognitive impairment through self-report or dichotomised quantitative scales. Previous studies focus on the first year of the COVID-19 pandemic, prior to introduction of vaccination and emerging variants.

**Added value of this study**

We report quantitatively on cognitive impairment following SARS-CoV-2 infection, from a large dataset of 4000 individuals with and without test-confirmed SARS-CoV-2 infection and a range of associated symptom durations, with mostly community-based cases. Importantly, we undertook two rounds of cognitive testing, allowing longitudinal tracking of cognitive performance and testing for deficits up to two years since infection.

**Implications of all the available evidence**

This study adds to existing evidence of cognitive deficits following SARS-CoV-2 infection, but finds important exceptions. At initial testing in mid-2021, cognitive deficits are not found for individuals who self-report as feeling recovered from COVID-19, even for those with longest symptom duration. In follow-up testing in mid-2022, we find that deficits appear persistent for those with earlier infections and ongoing symptoms, consistent with previous smaller studies. Further work should monitor those experiencing persistent cognitive impairment.

## Introduction

Persistent cognitive impairment and cognitive deficits after SARS-CoV-2 infection in comparison to individuals without infection have been reported from both subjective self-reported survey and objective assessments of cognitive functioning.^1^, ^2^, ^3^ Effects are similar to other infections.^4^^,^^5^ Studies using objective assessment to quantify the magnitude of cognitive deficits found deficits increased with severity of illness during the acute phase, with deficits among individuals requiring respiratory support or mechanical ventilation similar in magnitude to ageing 20 years from 50 to 70 years.^6^^,^^7^ A UK Biobank study comparing magnetic resonance images and objective cognitive tests recorded before and after SARS-CoV-2 infection revealed structural brain changes and longitudinal decline in cognitive performance,^8^ while markers of brain injury have been found in hospitalised COVID-19 patients.^9^

In addition to cognitive impairment in hospitalised individuals, cognitive impairment has also been reported in individuals with long-term and/or ongoing symptoms following SARS-CoV-2 infection, referred to most commonly as long COVID (clinical definitions and terms vary but generally refer to symptoms associated with SARS-CoV-2 infection which persist for more than 4 or 12 weeks since infection).^1^^,^^3^^,^^10^^,^^11^ While 17% of a UK cohort of hospitalised individuals met criteria of cognitive impairment from objective assessment at 6 months since hospital discharge,^12^ the UK Office for National Statistical estimated in January, 2023 that 1.0 million (52%) and 771,000 (39%) of 2.0 million individuals in the UK with self-reported long COVID (symptoms persisting for more than four weeks since infection) were experiencing difficulty concentrating and memory loss or confusion respectively.^13^ Ongoing symptoms experienced by individuals with long COVID are associated with difficulties in daily functioning, reduced ability to work, lower mental health and wellbeing, and lower self-reported quality-of-life.^14^, ^15^, ^16^, ^17^, ^18^ As well as effects on current functioning, SARS-CoV-2 infection may accelerate cognitive decline with age. A large-scale international study using electronic healthcare records to identify cases found increased risk of cognitive deficit and dementia persisting for at least two years after recorded COVID-19 diagnosis in comparison to other respiratory infections.^19^

Previous studies using objective cognitive assessments have mostly examined small, hospitalised cohorts, used dichotomised classifications rather than quantitative scales of cognitive impairment, focussed on individuals infected in the first year of the pandemic (i.e., before vaccination), with generally short follow-up (typically 6–12 months since infection).^1^, ^2^, ^3^ While studies have shown neurological deficits following SARS-CoV-2 infection, including in cohorts with long COVID, to our knowledge no studies have analysed a cohort with a range of symptom durations for both SARS-CoV-2 positive cases and negative controls, to examine the effects of both SARS-CoV-2 infection and symptom duration. Furthermore, few studies have looked longitudinally at cognitive trajectories of individuals and whether recovery relates to cognitive performance.

In this study, we used a validated cognitive assessment tool, with prospective self-report symptom assessment, and retrospective reflective survey data from a large UK voluntary cohort, the COVID Symptom Study Biobank, to address the following questions: 1) Is COVID-19 associated with cognitive performance? 2) Do symptom duration and ongoing symptoms affect any observed associations between COVID-19 and cognitive performance? 3) Do any associations between COVID-19 and cognitive performance change over time? We hypothesise that longer COVID-19 symptom duration has larger detriments to cognitive performance, while individuals reporting recovery from COVID-19 will show reduced cognitive deficits in comparison to those with ongoing symptoms.

## Methods

### COVID symptom study biobank cohort

Study participants were volunteers from the COVID Symptom Study Biobank (CSSB) United Kingdom cohort, approved by Yorkshire & Humber NHS Research Ethics Committee Ref: 20/YH/0298. Individuals were recruited to the CSSB via the COVID Symptom Study (CSS, later renamed ZOE Health Study) launched in the UK on March 24, 2020, approved by the King's College London Ethics Committee LRS-19/20–18210. All data were collected with informed consent obtained online. CSS study participants joined voluntarily after mass media campaigns to the general population from March 2020 onwards, without invitation via download from smartphone app stores. Participants were encouraged to enrol before they had symptoms or infection, to track development of symptoms across the pandemic. After registering with the CSS app, participants self-reported demographic information, symptoms potentially suggestive of COVID-19, any SARS-CoV-2 testing and results, and any vaccinations. CSS study participant composition, has been described in previous reports,^20^^,^^21^ and overrepresents middle age groups, female sex and individuals living in less deprived, more affluent areas in comparison to the general UK population. CSS participants from across the UK were invited to join the CSSB by email in October to November, 2020 and May, 2021.

CSSB invitation targeted five groups of regular CSS users with different SARS-CoV-2 infection statuses and associated symptom (illness) durations at the time of invitation. Case group 1 comprised individuals with positive SARS-CoV-2 test but no associated symptoms (asymptomatic COVID). Case group 2 comprised individuals with positive SARS-CoV-2 test and between one and 13 days of associated symptoms (short COVID). Case group 3 comprised individuals with positive SARS-CoV-2 test and at least 28 days of associated symptoms (long COVID). Control group 1 comprised individuals with negative SARS-CoV-2 test and at least 28 days of symptoms at the time of the test (long non-COVID). Control group 2, “healthy controls”, comprised individuals with negative SARS-CoV-2 test associated with one to maximum three consecutive days of illness at the time of the test, with low symptom burden (three symptoms or fewer; healthy non-COVID). Invited individuals were matched by Euclidian distance for age, sex and body mass index (BMI) across groups. Due to this targeted approach designed to give five equally sized groups, cohort composition is not representative of population prevalence of COVID-19 and long COVID. After consent, all participants were invited to give blood samples that were tested for anti-Nucleocapsid and anti-Spike SARS-CoV-2 antibodies.

8357 CSSB participants were invited to participate in a first round cognitive testing on July 12, 2021. Participants (whether participating in the first round or not) were also invited to a second testing round on April 28, 2022.

### Data collection

#### Cognitive assessment

Cognitive assessment was performed using the “Cognitron” online platform https://www.cognitron.co.uk/. In addition to studies of both hospitalised and community cases of COVID-19,^6^^,^^7^ cognitive batteries using the same platform have previously been shown to be sensitive to cognitive impairment in early Alzheimer's disease, cognitive decline in individuals with mild behavioural impairment (an at-risk state for dementia), cognitive function in older adults with high vs. low autistic traits, and “brain training” task repetition.^22^, ^23^, ^24^, ^25^, ^26^ Cognitive impairments following traumatic brain injury measured using the platform have also been found to correlate with corresponding brain networks measured by MRI.^27^ Although participants in this study performed testing remotely in unsupervised conditions, where differences in test environment may contribute to variation, cognitive deficits among individuals hospitalised with COVID-19 were previously found to be consistent between studies where data was collected under unsupervised and supervised conditions.^6^^,^^7^

In both rounds of assessment, participants undertook 12 cognitive tasks assessing different cognitive domains, including working memory, attention, reasoning, and motor control. Accuracy, average within-task reaction time, and variation in within-task reaction time metrics were extracted for each task and participant. Cognitive domain tested, performance metrics and transformation methods for each task are given in Table S1.

Concurrently with cognitive testing through the same online platform, participants completed the following assessments: Patient Health Questionnaire-2 (PHQ-2) and Generalised Anxiety Disorder-7 (GAD-7) assessments of depression and anxiety symptoms,^28^^,^^29^ Chalder Fatigue Scale (CFS),^30^^,^^31^ and Work and Social Adjustment Scale (WSAS) assessing functional impairment.^32^ The PHQ-4 measure of psychological distress was generated from the PHQ-2 and GAD-7.^33^ During second testing round only, individuals were also asked to report their highest level of educational attainment, as this is known to affect task performance.

#### Other data sources

The following variables were derived from self-reported data at registration with the CSS app: age, biological sex, ethnicity, number of physical health conditions (from asthma, cancer, diabetes, heart disease, kidney disease, lung disease), weight and height (from which BMI was derived), frailty (from the PRISMA-7 scale),^34^ local area deprivation (Index of Multiple Deprivation, IMD),^35^ and UK geographic region (from residential address data). Number of physical health conditions was grouped into a categorical variable with categories of 0, 1, and 2 or more conditions. Age, ethnicity, height, weight, and residential address were re-collected at time of consent to join CSSB and superseded data collected in the CSS app. SARS-CoV-2 testing data, symptoms data and whether individuals presented to hospital over the course of any illness were collected from the CSS app using ExeTera software (extraction date: 30 May, 2022).^36^ Number of mental health conditions was measured from self-reported diagnoses of 16 conditions collected in a February, 2021 CSS questionnaire, and grouped into a categorical variable with categories of 0, 1, 2, and 3 or more conditions. SARS-CoV-2 antibody testing results were generated from blood samples collected after consent at King's College London using previously reported methods.^37^ Self-perceived recovery from COVID-19 was collected in May–June, 2021 shortly before cognitive testing Round 1, as part of the CSSB “Effects of the Coronavirus Disease (COVID-19) pandemic on life in the UK” questionnaire.

A “COVID-19 group” exposure variable was derived as a composite combining SARS-CoV-2 test result (infection status) and duration of symptoms associated with the test (symptoms starting within a 14 day window either side of the positive or negative antigen test or more than 14 days before an antibody test), adapting methodology from a previous report (criteria provided in Supplementary information section S2 and visualised in Fig. S1).^21^ COVID-19 groups at both Round 1 and 2 of cognitive assessment were derived based on SARS-CoV-2 test and symptom data up to date of invitation to Round 1 and Round 2 respectively, allowing for changes in COVID-19 group between rounds due to new infection and/or symptoms. Symptom duration estimates were categorised based on COVID-19 clinical case definitions recommended in NICE guidelines on managing the long-term effects of COVID-19^10^: asymptomatic, symptoms up to 4 weeks (analogous to NICE “acute COVID-19”), symptoms between 4 and 12 weeks (analogous to NICE “ongoing symptomatic COVID-19”), and symptoms for 12 weeks or more (analogous to NICE “post-COVID-19 syndrome”). Equivalent symptom duration groupings associated with both negative and positive tests were used to give a total of 8 categories for COVID-19 group.

### Inclusion criteria

For all analyses, inclusion criteria were complete age, sex, ethnicity and area of residence data, and sufficient self-reported SARS-CoV-2 test results and symptom assessments logged on the CSS app at the time of invitation to Round 1 cognitive assessment to derive and assign a “COVID-19 group” described above. Full completion of all cognitive tasks was further required for generation of composite scores used as primary outcomes.

### Statistical analysis

#### Generation of inverse participation weights

Preliminary analysis of cognitive testing participation rates (including loss to follow-up between Round 1 and 2 of cognitive testing) showed variation with our primary exposures of interest (SARS-CoV-2 infection status and associated symptom duration). Knowing this may act as a potential source of response and collider bias (as demonstrated in other COVID-19 research^38^), logistic regression models were run to predict participation in cognitive assessments and generate weights of inverse probability of participation. Weights generated from these models were included in subsequent analyses in attempt to account for bias. Further details of weight generation are given in Supplementary information section S3.

#### Cognitive assessment data processing

For the small number of individuals who completed a task more than once within the same testing round (due to multiple attempts to complete the cognitive assessment), task metrics were taken for the earliest attempt to prevent learning curve effects. Reaction time metrics more than three standard deviations from the mean were winsorised to reduce effects of outliers as done previously.^6^ Transformation methods were applied to each task metric (square, cube, square root or logarithmic), to minimise skewness of distributions (Table S1). Following transformation, metrics for each task were standardised into z-scores representing number of standard deviations from the mean.

#### Principal component analysis

To reduce dimensionality and assess global cognitive performance across a broad range of domains, as in previous reports,^6^^,^^7^ principal component analysis (PCA) was used to generate composite scores for accuracy, average reaction time, and reaction time variation across all cognitive tasks. PCA was performed separately on Round 1 and Round 2 datasets, with the number of components selected using a previously reported method.^39^ Principal component composite scores were converted to standardised z-scores for use in subsequent analyses.

#### Proposed causal pathways: directed acyclic graphs

**Fig. 1:**
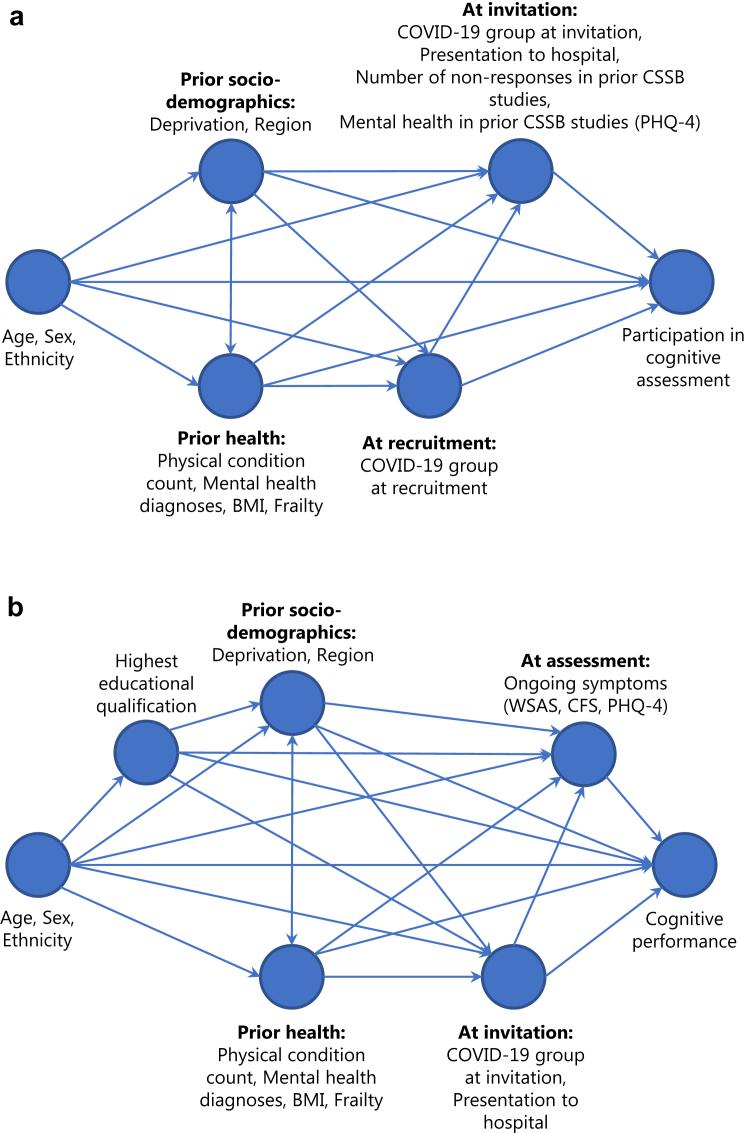
**Directed acyclic graph describing hypothesised causal pathways.** Proposed directed acyclic graphs (DAGs) used to generate minimal adjustment variable sets for estimation of the total causal effect of variables on outcomes of participation in cognitive assessment (a) and cognitive performance (b). DAGs are structured approximately in order of data generation from left to right.

Using a causal inference approach to estimate the individual effects of various exposure variables on both participation in cognitive assessment and cognitive performance, directed acyclic graphs (DAGs) describing proposed relationships between observed variables were constructed using dagitty software http://www.dagitty.net/dags.html (Fig. 1, full code given in Supplementary information section S5).^40^ Hypothesised relationships between variables shown in DAGs were informed by previously observed associations wherever possible.

Our primary outcome variable was the first principal component composite score for accuracy across all cognitive tasks generated in PCA. Our secondary outcome variables were: PCA composites for average reaction time, PCA composites for reaction time variation, and accuracy, average reaction time and reaction time variation for the individual tasks that comprised the cognitive testing battery.

To estimate the total causal effect of each exposure listed on the outcomes of interest (i.e., participation and cognitive performance), separate models were run for each exposure. Variables that are hypothesised as confounding the causal relationship between the exposure and outcome in DAGs were included as adjustments in models. Sets of adjustment variables for each combination exposure and outcome variable are tabulated in Table S2. In this way, the so-called “Table 2 fallacy” (misinterpretation of effect estimates of adjustment variables often presented in multivariable model results as if they were the exposure of interest—in which case they may require a different adjustment set) can be avoided.^41^ For analyses of individuals who participated in Round 1 cognitive testing, local area deprivation and UK geographic region were used as proxy variables for educational attainment level, which was only collected in Round 2 of assessment. For analyses of individuals that completed Round 2 or both Round 1 and 2, educational attainment level collected at Round 2 was used with the assumption that educational attainment level was constant.

#### Regression models

The total effects of exposure variables on participation in cognitive testing were estimated using ordinary logistic regression models containing the outcome and exposure variable of interest, in addition to the appropriate adjustment set (Fig. 1).

The total effects of exposure variables on cognitive performance metrics (accuracy, reaction time and variation in reaction) were estimated using ordinary least squares linear regression models containing the outcome and exposure variable of interest, in addition to the appropriate adjustment set (Table S2). Variables were entered without hierarchy, in a single-level model. A separate model was run for each exposure variable of interest, for each outcome variable (accuracy, reaction time and variation in reaction time).

To estimate the longitudinal change over time in the outcome of cognitive accuracy between Round 1 and Round 2 of testing, we adopted a “follow-up adjusted for baseline” approach, as outlined in guidance on estimating change over time by Tennant et al.^42^ We hypothesised that the accuracy score outcome at Round 1 acted as a mediator in the relationship between COVID-19 exposure variables and the outcome at Round 2, due to the strength of prior evidence of the effect of COVID-19 on cognition, and because COVID-19 group data was generated up to several months before cognitive testing took place. Using this causal scenario, we included Round 1 accuracy score as an additional variable in models that tested the effect of COVID-19 group on Round 2 accuracy score. Using this approach, the model coefficient for COVID-19 group represents the direct effect of COVID-19 group on the change in cognitive performance between Round 1 and 2. This approach has been shown to be a less biased estimate of change over time in comparison to use of a “change score” as an outcome.^42^ For all models with the same outcome, p-values were adjusted for multiple testing using the Benjamini/Hochberg method.^43^

#### Software

Analyses were performed using python v3.8.8 and packages: numpy v1.20.1, pandas v1.2.4, statsmodels v0.12.2, scipy v1.6.2, scikit-learn v0.24.1, matplotlib v3.3.4, seaborn v0.11.1, factor_analyzer v0.4.0.

### Role of the funding source

The funders of the study had no role in the design of the study, data collection, data analysis, interpretation or writing of the report. All authors had full access to all data within the study. The corresponding authors had final responsibility for the decision to submit for publication.

## Results

### Sample characteristics

Of 8357 individuals invited to Round 1 and/or Round 2 of cognitive assessment, 7588 Round 1 invitees and 7198 Round 2 invitees met inclusion criteria for analysis (Fig. S2). Of these, 3335 completed all cognitive assessment tasks in Round 1, 2435 in Round 2, and 1768 in both rounds.

Participants in cognitive assessments were skewed towards middle age groups (Round 1: median = 57 years [IQR = 50–64], Round 2: median = 58 years [IQR = 51–64]), female sex (Round 1: 81%, Round 2: 82%), white ethnicity (Round 1: 96%, Round 2: 97%), living in lower deprivation neighbourhoods (IMD Quintile 5, least deprived 20%, Round 1: 35%, Round 2: 34%), reflecting CSSB cohort composition (Table 1).

**Table 1:** Sample characteristics.

Variable	Category	Invited to Round 1 & meeting inclusion criteria	Round 1	Round 2	Full completion of both Round 1 and 2
**TOTAL COUNT**		7588	3335	2435	1768
Age group (years)	18–30	166 (2.2%)	43 (1.3%)	23 (0.9%)	15 (0.8%)
	30–40	695 (9.2%)	214 (6.4%)	128 (5.3%)	80 (4.5%)
	40–50	1490 (19.6%)	539 (16.2%)	379 (15.6%)	258 (14.6%)
	50-60 (reference)	2572 (33.9%)	1183 (35.5%)	860 (35.3%)	622 (35.2%)
	60–70	2059 (27.1%)	1042 (31.2%)	804 (33.0%)	613 (34.7%)
	70–80	570 (7.5%)	293 (8.8%)	223 (9.2%)	168 (9.5%)
	≥80	36 (0.5%)	21 (0.6%)	18 (0.7%)	12 (0.7%)
Sex	Female (reference)	6032 (79.5%)	2697 (80.9%)	2001 (82.2%)	1445 (81.7%)
	Male	1556 (20.5%)	638 (19.1%)	434 (17.8%)	323 (18.3%)
Ethnicity	Asian/Asian British	61 (0.8%)	25 (0.7%)	14 (0.6%)	11 (0.6%)
	Black/Black British	27 (0.4%)	12 (0.4%)	5 (0.2%)	<5
	Mixed/Multiple	98 (1.3%)	41 (1.2%)	25 (1.0%)	19 (1.1%)
	Other	111 (1.5%)	44 (1.3%)	26 (1.1%)	18 (1.0%)
	White (reference)	7291 (96.1%)	3213 (96.3%)	2365 (97.1%)	1717 (97.1%)
Highest educational attainment level^a^	Data not available	4422 (58.3%)	1194 (35.8%)	0 (0.0%)	0 (0.0%)
	Postgraduate degree or higher	959 (30.3%)	658 (30.7%)	759 (31.2%)	557 (31.5%)
	Undergraduate degree (reference)	1169 (36.9%)	794 (37.1%)	900 (37.0%)	653 (36.9%)
	Less than undergraduate degree	994 (31.4%)	661 (30.9%)	740 (30.4%)	534 (30.2%)
	Other/Prefer not to say	44 (1.4%)	28 (1.3%)	36 (1.5%)	24 (1.4%)
Local area deprivation (IMD)	Quintile 1 (most 20% deprived areas)	470 (6.2%)	188 (5.6%)	138 (5.7%)	97 (5.5%)
	Quintile 2	1014 (13.4%)	413 (12.4%)	335 (13.8%)	235 (13.3%)
	Quintile 3 (reference)	1517 (20.0%)	682 (20.4%)	486 (20.0%)	359 (20.3%)
	Quintile 4	2010 (26.5%)	883 (26.5%)	640 (26.3%)	469 (26.5%)
	Quintile 5 (least 20% deprived areas)	2577 (34.0%)	1169 (35.1%)	836 (34.3%)	608 (34.4%)
SARS-CoV-2 test result^b^	Negative (reference)	3453 (45.5%)	1598 (47.9%)	809 (33.2%)	854 (48.3%)
	Positive	4135 (54.5%)	1737 (52.1%)	1626 (66.8%)	914 (51.7%)
COVID-19 group^b^	SARS-CoV-2 Negative, Asymptomatic (reference)	1139 (15.0%)	641 (19.2%)	220 (9.0%)	388 (21.9%)
	SARS-CoV-2 Negative, Symptom duration <4 weeks	919 (12.1%)	379 (11.4%)	262 (10.8%)	190 (10.7%)
	SARS-CoV-2 Negative, Symptom duration 4–12 weeks	977 (12.9%)	372 (11.2%)	218 (9.0%)	175 (9.9%)
	SARS-CoV-2 Negative, Symptom duration ≥12 weeks	418 (5.5%)	206 (6.2%)	109 (4.5%)	101 (5.7%)
	SARS-CoV-2 Positive, Asymptomatic	706 (9.3%)	256 (7.7%)	165 (6.8%)	122 (6.9%)
	SARS-CoV-2 Positive, Symptom duration <4 weeks	1601 (21.1%)	589 (17.7%)	658 (27.0%)	279 (15.8%)
	SARS-CoV-2 Positive, Symptom duration 4–12 weeks	985 (13.0%)	397 (11.9%)	406 (16.7%)	209 (11.8%)
	SARS-CoV-2 Positive, Symptom duration ≥12 weeks	843 (11.1%)	495 (14.8%)	397 (16.3%)	304 (17.2%)
Presented to hospital during symptomatic period^b^	No (reference)	7003 (92.3%)	3054 (91.6%)	2172 (90.7%)	1594 (90.2%)
	Yes	585 (7.7%)	281 (8.4%)	223 (9.3%)	174 (9.8%)
Weeks between cognitive assessment and symptom start/test date^b^		53.0 (31.0, 67.0)	42.0 (29.0, 62.0)	78.0 (38.0, 103.0)	77.0 (40.0, 101.0)
Symptom start date/Test date^b^	Q1 January–March, 2020	933 (12.3%)	385 (11.5%)	263 (10.8%)	187 (10.6%)
	Q2 April–June, 2020	2497 (32.9%)	879 (26.4%)	511 (21.0%)	332 (18.8%)
	Q3 July–September, 2020	1065 (14.0%)	469 (14.1%)	232 (9.5%)	179 (10.1%)
	Q4 October–December, 2020	1746 (23.0%)	830 (24.9%)	500 (20.5%)	386 (21.8%)
	Q1 January–March, 2021	603 (7.9%)	319 (9.6%)	129 (5.3%)	102 (5.8%)
	Q2 April–June, 2021	712 (9.4%)	433 (13.0%)	155 (6.4%)	126 (7.1%)
	Q3 July–September, 2021	32 (0.4%)	20 (0.6%)	78 (3.2%)	57 (3.2%)
	Q4 October–December, 2021	0	0	196 (8.0%)	141 (8.0%)
	Q1 January–March, 2022	0	0	305 (12.5%)	212 (12.0%)
	Q2 April–June, 2022	0	0	66 (2.7%)	46 (2.6%)

Characteristics of participants in Round 1 and/or Round 2 of cognitive assessment. Counts and proportions are presented apart from weeks between cognitive assessment and symptom start/test date, where median and interquartile range is given.

a Education level was collected as part of Round 2 of cognitive assessment.

b For variables relating to COVID-19 history, counts are given at the time of invitation to the relevant round (counts at invitation to Round 1 for column detailing participants in both rounds). Other variables were collected or derived from data collected at recruitment or prior to analyses.

The largest COVID-19 groups were individuals with a positive test and symptoms of up to four weeks, Round 1: 18%, Round 2: 27% (meeting NICE “acute-COVID-19” criteria^10^) and individuals with a positive test and symptoms lasting 12 or more weeks, Round 1: 15%, Round 2: 16% (meeting NICE “post-COVID-19 syndrome” criteria). The number of individuals in the “healthy control” COVID-19 group, with only negative test(s) and no associated symptoms, decreased between Rounds 1 and 2 because of SARS-CoV-2 infection (Round 1: 19%, Round 2: 9%). Sample characteristics stratified by COVID-19 group are given in Table S3. In line with CSSB recruitment that was based on symptom duration in 2020, almost all individuals with positive tests and associated symptoms of 12 or more weeks were infected in 2020 (Round 1: 98%, Round 2: 91%).

### Participation analysis

Logistic regression analysis of factors associated with participation in cognitive assessments revealed multiple associations, with individuals with a higher number of prior non-responses to other CSSB studies, more recently recruited individuals, older age groups, and female sex individuals more likely to participate (Fig. S3, Fig. S4, Table S4). We found very similar patterns of association with participation for partial as well as full completion of cognitive assessments (not presented). Predictive performance of models used to generate weights of inverse probability of participation for subsequent analyses was moderate for Round 1 participation (AUC-ROC = 0.72), and good for Round 2 participation (AUC-ROC = 0.82) and participation in both Round 1 and 2 (AUC-ROC = 0.87) (Table S5).

### Principal component analysis of cognitive performance

Separate principal component analyses (PCA) of accuracy, average reaction time, and intra-task reaction time variation metrics from Round 1 and 2 of cognitive assessment produced three, two, and three components with an eigenvalue greater than one respectively (Table S6 and Table S7). Very similar loadings and variance explained were found for the first and second principal components for each performance metric across both rounds of testing. As a result, loadings from Round 1 PCA were used to generate composite scores for first and second principal components for both Round 1 and 2, for each of accuracy, average reaction time, and reaction time variation. The first principal component from PCA of task accuracy scores represented higher accuracy across all tasks (26% of variance). The first component from average task reaction time PCA represented larger average reaction time (44% of variance), and first component of variation in reaction time PCA represented larger reaction time variation (25% of variance). First component scores were used as the primary outcomes of interest in subsequent analyses.

### Cross-sectional cognitive performance

**Fig. 2:**
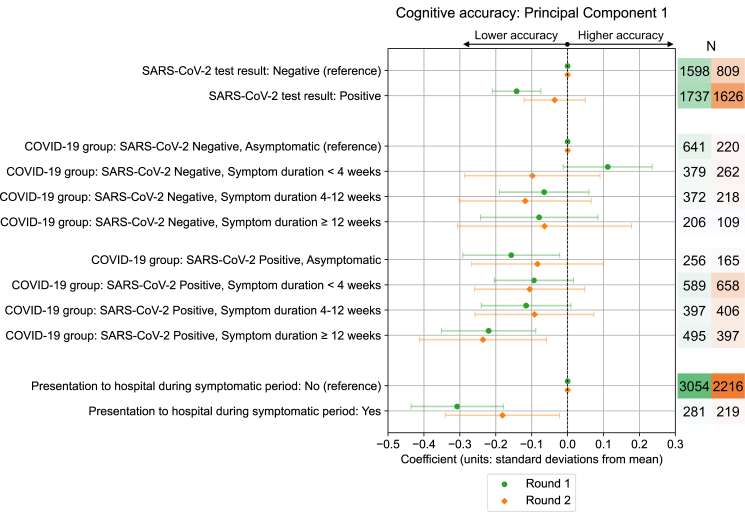
**Association between COVID-19 exposures and cognitive accuracy scores**. Standardised coefficients (number of standard deviations from mean) with 95% confidence intervals from multivariable ordinary least squares linear regression models testing association between COVID-19 related exposures and cognitive accuracy PCA 1st component standardised scores. Results are from Round 1 and Round 2 of cognitive testing, among all individuals who completed either round of testing. Results for each exposure variable presented originate from separate models that use distinct adjustment variable sets determined from the proposed DAG for cognitive performance (Test result—Age, BMI, Deprivation, Education [Round 2 model only], Ethnicity, Frailty, Mental health condition count, Physical health condition count, Presentation to hospital, Region, Sex, Symptom duration; COVID-19 group – Age, BMI, Deprivation, Education [Round 2 model only], Ethnicity, Frailty, Mental health condition count, Physical health condition count, Presentation to hospital, Region, Sex; Presentation to hospital – Age, BMI, COVID-19 group at invitation, Deprivation, Education [Round 2 model only], Ethnicity, Frailty, Mental health condition count, Physical health condition count, Region, Sex).

At Round 1 of cognitive testing, lower composite cognitive accuracy scores, or “cognitive deficits”, were found for the SARS-CoV-2 positive COVID-19 group with ≥12 weeks symptom duration (β = −0.22 standard deviations from mean [SDs], 95% CI: −0.35, −0.09, adjusted p = 0.0045), relative to the SARS-CoV-2 negative COVID-19 group with no symptoms (Fig. 2). The scale of deficit was similar to the effect size of presentation to hospital during illness (β = −0.31 SDs, 95% CI: −0.44, −0.18, adjusted p < 0.0001), or of age group differences of 10 years within the same sample, e.g., age 60–70 years vs. 50–60 years (β = −0.21 SDs, 95% CI: −0.30, −0.13, adjusted p < 0.0001), or age 40–50 years vs. 50–60 years (β = +0.24 SDs, 95% CI: 0.15, 0.34, adjusted p < 0.0001) (Fig. S5). While controlling for symptom duration, an overall deficit was observed for individuals with positive SARS-CoV-2 tests compared to individuals testing negative (β = −0.14 SDs, 95% CI: −0.21, −0.07, adjusted p = 0.00026). Cognitive deficits in SARS-CoV-2 positive COVID-19 groups remained present in the subset of individuals who completed both Round 1 and 2 of assessment, for whom educational attainment data was available and included as an additional adjustment variable (Fig. S6). There was no evidence of cognitive deficits in individuals in symptomatic SARS-CoV-2 negative COVID-19 groups. Unadjusted cognitive accuracy scores, split by SARS-CoV-2 test result and COVID-19 group are given in Fig. S7 and Fig. S8.

At Round 2 of testing, lower cognitive accuracy scores were again observed for the SARS-CoV-2 positive COVID-19 group with ≥12 weeks symptom duration, and individuals who presented to hospital during illness. However, effect sizes and/or strength of associations (wider confidence intervals and larger p-values) with SARS-CoV-2 infection were generally reduced in comparison to Round 1. We note that associations between cognitive accuracy and COVID-19 exposures were also present in models where weights were winsorised (not presented), capping the 5% smallest and largest weight values at the 5th and 95th percentile values respectively, to test the dependency of findings on the most extreme weight values.

Considering the effect of COVID-19 exposures on accuracy in individual tasks in Round 1 (Fig. S9), tasks with most consistent evidence of lower accuracy scores by SARS-CoV-2 positive groups were those concerning episodic visual memory, in tasks testing immediate and delayed recall of objects, and visual attention and processing speed in the ‘target detection’ task where participants found and selected target shapes within an evolving grid of shapes. Evidence of deficits at an individual task level for SARS-CoV-2 positive groups was more variable for other tasks that tested working memory, spatial planning and mental manipulation, motor control, and semantic reasoning (Table S1).

Further multivariable models found several other exposures were associated with lower cognitive accuracy for both principal components composites and individual task scores, namely older age, lower educational attainment level, living in certain UK regions, and areas of higher deprivation (used as socio-economic indicators in models of all Round 1 participants for whom educational data was incomplete), BMI in the underweight, overweight or obese range, having one or more physical health conditions (from asthma, cancer, diabetes, heart disease, kidney disease, lung disease), and above threshold scores indicative of poorer mental health, high fatigue levels, and functional impairment in assessments reported contemporaneously with cognitive assessments (Fig. S5 and Fig. S10). Multivariable models testing associations with within-task average reaction time and reaction time variation found higher within-task variation in reaction time for SARS-CoV-2 positive individuals with ≥12 weeks symptom duration in Round 1 (Fig. S11), but no evidence of differences in average reaction time among SARS-CoV-2 positive groups (Fig. S12).

### Role of ongoing symptoms

**Fig. 3:**
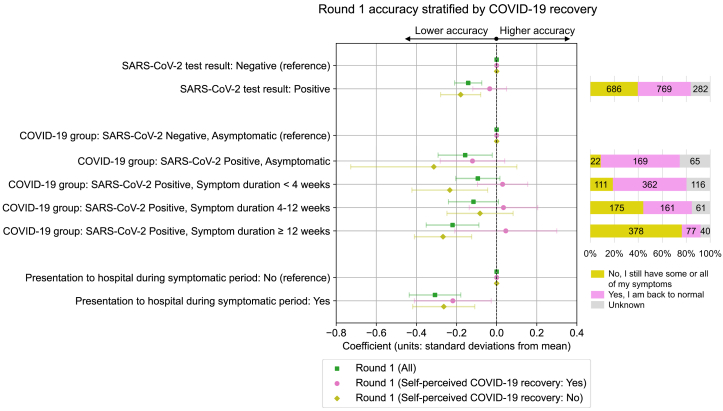
**Association between COVID-19 exposures and Round 1 cognitive accuracy scores, stratified by self-perceived recovery from COVID-19**. Standardised coefficients (number of standard deviations from mean) with 95% confidence intervals from multivariable ordinary least squares linear regression models testing association between COVID-19 related exposures and cognitive accuracy PCA 1st component standardised scores. Results are from all individuals who completed Round 1 of cognitive testing, stratified by response to the question “Thinking about the last or only episode of COVID-19 you have had, have you now recovered and are back to normal?“, with cross-tabulations presented for SARS-CoV-2 positive individuals. Results for each exposure variable presented originate from separate models that use distinct adjustment variable sets determined from the proposed DAG for cognitive performance (Test result – Age, BMI, Deprivation, Ethnicity, Frailty, Mental health condition count, Physical health condition count, Presentation to hospital, Region, Sex, Symptom duration; COVID-19 group – Age, BMI, Deprivation, Ethnicity, Frailty, Mental health condition count, Physical health condition count, Presentation to hospital, Region, Sex; Presentation to hospital—Age, BMI, COVID-19 group at invitation, Deprivation, Ethnicity, Frailty, Mental health condition count, Physical health condition count, Region, Sex).

To test the role of ongoing symptoms in the observed cognitive deficits, further models testing associations with Round 1 cognitive accuracy were run. Firstly, the role of self-perceived recovery from COVID-19 was tested by stratifying SARS-CoV-2 positive individuals by their response to the question “Thinking about the last or only episode of COVID-19 you have had, have you now recovered and are back to normal?” in a separate CSSB survey undertaken shortly before Round 1, completed by 84% (N = 1455/1737) of SARS-CoV-2 positive individuals at Round 1 (Fig. 3).

After stratification, cognitive deficits were not observed among SARS-CoV-2 positive individuals (N = 769) who responded “Yes, I am back to normal”, both for individuals with ≥12 weeks symptoms (β = +0.05 SDs, 95% CI: −0.21, 0.30, adjusted p = 0.86) and overall while controlling for symptom duration (β = −0.03 SDs, 95% CI: −0.12, 0.05, adjusted p = 0.72). Conversely, overall deficits in SARS-CoV-2 positive were increased compared to the unstratified sample for the 686 individuals who responded “No, I still have some or all of my symptoms” (β = −0.18 SDs, 95% CI: −0.28, −0.08, adjusted p = 0.0032). However, self-perceived COVID-19 recovery among SARS-CoV-2 positive individuals was highly correlated with symptom duration (Fig. S14, Spearman correlation coefficient for weeks of symptoms vs. proportion recovered, r = −0.83, p < 0.0001).

Individuals who presented to hospital during illness showed a similar scale of cognitive deficits independent of self-perceived recovery from COVID-19. A small proportion of individuals (22/256, 9%) who did not report any symptoms prospectively via the CSS app at the time of their SARS-CoV-2 infection and so were considered as asymptomatic reported as “still having some or all of their symptoms” when asked retrospectively about their recovery from COVID-19 infection.

Further analyses used mental health, fatigue, and functional impairment assessments collected contemporaneously with cognitive assessment to test mediation of cognitive deficits by specific symptom types. Associations between COVID-19 group and cognitive accuracy in mediation models showed reduced effect sizes and 95% confidence levels which crossed β = 0 for some groups in both Round 1 and Round 2 (Fig. S13). Results suggest partial mediation by these symptom types on the effect of COVID-19 group on cognitive accuracy, with mediation effects larger for longer symptom duration groups.

### Longitudinal change in cognitive accuracy between rounds

**Fig. 4:**
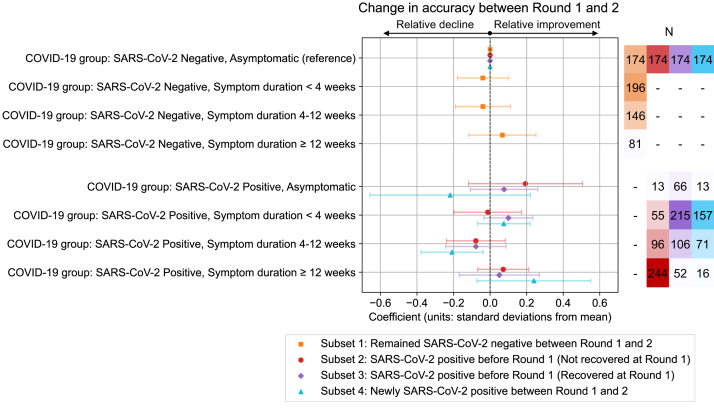
**Estimated change in cognitive accuracy between rounds of cognitive testing relative to change observed in reference groups.** Standardised coefficients (number of standard deviations from mean) from multivariable ordinary least squares linear regression models testing association between COVID-19 group and cognitive accuracy PCA 1st component standardised scores. Models are presented for subsets of individuals who completed both rounds of testing, selected based on change in COVID-19 group between Round 1 and 2 and self-perceived COVID-19 recovery at Round 1. Models included Round 1 cognitive accuracy score as a mediator in order for coefficients to estimate change in accuracy between rounds due to COVID-19 group, in addition to the following adjustment variables: Age, BMI, Deprivation, Education, Ethnicity, Frailty, Mental health condition count, Physical health condition count, Presentation to hospital, Region, Sex.

Finally, to estimate the effect of COVID-19 group on change in cognitive accuracy between rounds of testing, Round 1 cognitive accuracy was included in models testing association between Round 2 cognitive accuracy and COVID-19 exposures, to control for Round 1 performance and isolate change in accuracy between rounds (Fig. 4).

Models were run on four subsets of 1768 individuals who completed both rounds of cognitive testing selected based on change in COVID-19 group between Round 1 and 2 and self-perceived COVID-19 recovery at Round 1. Each subset included 174 individuals in the SARS-CoV-2 negative, asymptomatic COVID-19 group at both cognitive assessment rounds to act as the comparison group, in addition to: Subset 1–431 individuals who remained SARS-CoV-2 negative between rounds; Subset 2–408 individuals who tested SARS-CoV-2 positive *prior* to Round 1 and self-reported as *not recovered* from COVID-19 prior to Round 1; Subset 3–439 individuals who tested SARS-CoV-2 positive *prior* to Round 1 and self-reported as *recovered* from COVID-19 prior to Round 1; Subset 4–257 individuals who first tested SARS-CoV-2 positive *between* Round 1 and 2. There was no evidence of change in cognitive accuracy between rounds in the Subset 1 individuals who remained SARS-CoV-2 negative (relative to change over time in the negative, asymptomatic reference group). There was also no evidence of change in the individuals with positive tests prior to Round 1 in Subsets 2 and 3, with no differences in change between subsets according to self-perceived recovery from COVID-19. Among Subset 4 individuals who first tested SARS-CoV-2 positive between rounds, while individuals with 4–12 weeks symptom duration showed a relative decline between rounds (β = −0.21 SDs, 95% CI: −0.38, −0.04, adjusted p = 0.041), effects for other positive groups were weaker and inconsistent in size and direction, suggesting no clear pattern with symptom duration and little evidence of change over time.

## Discussion

Our results partially support the hypothesis that those with community-based SARS-CoV-2 infection show cognitive deficits in performance accuracy relative to non-infected individuals, but only among groups with ≥12 weeks symptom duration from prospective symptom logging and/or self-reporting as not recovered and “back to normal” following infection. For these individuals with detectable deficits at initial testing, longitudinal follow-up showed deficits persisted at almost two years since infection.

CSSB cohort design with SARS-CoV-2 negative and positive groups across a range of symptom durations enabled effects of infection and symptom duration to be disentangled. At Round 1 testing, lower cognitive task accuracy scores were observed for individuals with positive vs. negative SARS-CoV-2 infection status while controlling for symptom duration, with largest deficits seen for those with ≥12 weeks of associated symptoms (Fig. 2). Such individuals may self-define as having “long COVID” and meet NICE “Post-COVID-19 syndrome” and WHO “Post COVID-19 condition” definitions.^10^^,^^11^ The deficits in composite task accuracy scores were comparable in scale to the effect of presentation to hospital during illness, an increase in age of approximately 10 years, or exhibiting mild or moderate symptoms of psychological distress, but smaller than other effects such as lower educational attainment or above threshold fatigue levels (Fig. S5). Conversely, we found no evidence of an effect of SARS-CoV-2 infection on average reaction time during tasks (Fig. S10) in contrast to observations of individuals who received critical care for COVID-19 using the same assessment platform,^7^ and a relatively weak effect on variation in reaction time (Fig. S11). This is a reassuring finding given the importance of processing speed within cognition and extensive relationships with outcomes such as frailty, dementia and later mortality.^44^, ^45^, ^46^

Importantly, we found no detectable impairment among people who reported as feeling recovered and “back to normal” after their COVID-19 illness, even among individuals who experience long-term symptoms of ≥12 weeks (Fig. 3). Similarly, presence of ongoing symptoms of psychological distress, fatigue, and functional impairment at the time of cognitive testing partially mediated observed cognitive deficits, suggesting that reductions in these symptoms are elements (but not the whole) of recovery and associated cognitive deficit. These findings are similar to a previous smaller study which found higher cognitive performance for 42 individuals who self-reported as recovered vs. 117 with ongoing symptoms.^47^ However, recovery rate was highly correlated with symptom duration (Fig. S14), with a recovery rate of only 17% (N = 77/455) for those with ≥12 weeks symptom duration at 38 weeks (IQR: 31–63) since infection. Similarly low recovery rates at 12 months since infection have been reported in other cohorts following individuals with long COVID (15%) and after hospitalisation (29%).^17^^,^^48^

Longitudinal follow-up at a follow-up time of 9 months found no evidence of change in cognitive accuracy (neither improvement nor decline) for individuals who had SARS-CoV-2 infection and reported as not recovered before Round 1 (380/408, 93% prior to vaccination), with deficits persisting at almost two years since infection (median: 84 weeks, IQR: 74, 108).

Conversely, in an opportunistic analysis of individuals who were recruited with SARS-CoV-2 negative statuses but had first infections between rounds of cognitive assessment, we found much less convincing evidence of cognitive sequelae for these later COVID-19 infections. Such infections occurred after vaccination against SARS-CoV-2 (>99% of 257) and skewed towards shorter durations than infections before Round 1, which may reflect our sampling strategy, as well as the reduced likelihood of long COVID (illness duration ≥4 weeks) for more recent delta vs. alpha and omicron vs. delta variants,^49^^,^^50^ and following vaccination.^20^

There are some limitations to our study. Certain data that would have informed the study were unavailable, such as information on prior neurovascular and neurodegenerative comorbidities, cognitive assessment data prior to SARS-CoV-2 infection (for most cases), or information on treatment or cognitive rehabilitation following SARS-CoV-2 infection. The battery of cognitive tasks used in this study were not exhaustive and further understanding of the effects of COVID-19 may be gleaned from full neuropsychological testing, which was not possible in a study of this scale. Despite efforts to address potential selection and participation biases, it is possible some remain. The generalisability of our findings is limited by CSSB cohort composition, which has lower proportions of males, racialised non-white ethnic groups, individuals without university-level education, and those living in more deprived areas than the UK population. As such, replication is needed in other populations. Finally, our study relied on voluntary prospective logging of symptoms and SARS-CoV-2 test results via a smartphone app to derive SARS-CoV-2 infection status and estimate symptom duration as well as retrospective survey responses. Both datasets may be imperfect and incomplete, leading to misclassification in some cases.

In summary, individuals with ≥12 weeks symptoms following SARS-CoV-2 infection in the first year of the pandemic had detectable deficits in cognitive accuracy. Those with ongoing symptoms at initial testing did not show cognitive recovery at follow-up 9 months later. The population infected in 2020 with ongoing symptoms, to whom this result is most likely to apply, is sizeable—UK Office for National Statistics estimated that as of January, 2023, 687,000 in the UK were experiencing self-reported long COVID (defined as having ongoing symptoms at more than 4 weeks since infection) after a first infection at least two years previously.^13^ The scale of deficits we observed may have detrimental impacts on quality-of-life and daily functioning at an individual level as previously reported,^14^ as well as socio-economic impacts on society more broadly due to both a reduced capacity to work and an increased need for support. With infrequent and inconsistent identification of long COVID in electronic health care records,^51^ this work calls for renewed efforts to identify those affected by ongoing symptoms following SARS-CoV-2 infection. Our results highlight the importance of assessing the *ongoing* element of long COVID definitions, which appears to be a better predictor of cognitive impairment due to COVID-19 than symptom duration. Future work needs to focus on trajectories and mechanisms of recovery from ongoing symptoms following COVID-19, as well as the long-term implications on individuals and society of the persistent cognitive deficits observed in this study.

## Contributors

CJS, RP, and NJC were primarily responsible for conceptualisation, with contribution from all authors. CJS, ED, and SO were responsible for funding acquisition. VB, PH, and AH were responsible for project administration. CJS, RP, NJC, VG, AH, PH, and CHS were responsible for methodology. NJC was responsible for formal analysis. NJC, RP, VB, PH, AH, KJD, and MHM were responsible for investigation. NJC was responsible for visualisation. VB, LC, LSC, NJC, JD, AH, PH, EK, BM, and CHS were responsible for data curation. NJC, CJS, VB, VG, AH, PH, RP, CHS, ED, and EM were responsible for writing the original draft. All authors contributed to review and editing of the manuscript. NJC and VB have verified the underlying data. All authors had full access to study data and were responsible for the decision to submit for publication.

## Data sharing statement

Access to data in the CSS Biobank is available to bona fide health researchers on application to the CSS Biobank Management Group. Further details are available online at https://cssbiobank.com/information-for-researchers including application forms and contact information. Analysis code used in this study are available openly on Github at https://github.com/nathan-cheetham/CSSBiobank_CognitiveAssessment. Anonymised COVID Symptom Study data are available to researchers to be shared with researchers according to their protocols in the public interest through Health Data Research UK (HDRUK) and Secure Anonymised Information Linkage consortium, housed in the UK Secure Research Platform (Swansea, UK) at https://web.www.healthdatagateway.org/dataset/fddcb382-3051-4394-8436-b92295f14259. For the purpose of open access, the author has applied a Creative Commons Attribution (CC BY) licence to any Author Accepted Manuscript version arising.

## Declaration of interests

NJC is supported by NIHR via their institution; CHS is supported by Alzheimer's Society via their institution and is Scientific Advisor to BrainKey; WRT is a part time employee H2 Cognitive Designs, who market the online testing platform used in this study; PJH is the Chief Executive and Co-founder of H2 Cognitive Designs LTD, who market the online testing platform used in this study; M. Modat reports funding support from UK Department of Health and Social Care, UKRI, EPSRC and Wellcome Trust via their institution; SO is supported by French National Research Agency, Wellcome Trust, EPSRC and UK Department of Health and Social Care; A. Hampshire is an owner/director of Future Cognition Ltd and co-owner and co-director of H2 Cognitive Designs, which provide cognitive assessment services and software for third parties; CJS is supported by UKRI, Wellcome Trust and Chronic Disease Research Foundation via their institution, and declares a consultancy contract with ZOE Ltd. All other authors make no disclosures.
